# Genome-wide identification of the ABC gene family in sweet potato and its expression profiles in response to iron, aluminum, zinc, and copper stresses

**DOI:** 10.3389/fpls.2026.1815302

**Published:** 2026-06-10

**Authors:** Fulu Gao, Yanhui Lin, Jinyang Zhao, Muhammad Shahid, Luqman Khan, Yujie Li, Xianming Zhou, Chengcheng Si

**Affiliations:** 1 School of Tropical Agriculture and Forestry, Sanya Institute of Breeding and Multiplication, Hainan University, Sanya, Hainan, China; 2 Institute of Food Crops, Hainan Academy of Agricultural Sciences/Hainan Key Laboratory of Crop Genetics and Breeding, Haikou, Hainan, China; 3 Hainan Seed Industry Laboratory, Sanya, Hainan, China

**Keywords:** ATP-binding cassette transporter, gene expression, genome-wide analysis, heavy metal stress, phylogenetic evolution, sweet potato

## Abstract

ATP-binding cassette (*ABC*) transporters constitute one of the largest gene families in plants. These encoded transmembrane proteins rely on energy provided by ATP hydrolysis to facilitate the directional transport of diverse substrates, such as heavy metal (HM) ions, secondary metabolites, and phytohormones, playing a pivotal role in plant responses to HM stress. In this study, a systematic identification and expression analysis of the *ABC* gene family in sweet potato (*Ipomoea batatas*) was conducted at the whole-genome level. We comprehensively characterized the physicochemical properties, structural features, chromosomal distribution, and tissue-specific expression patterns of *IbABC* genes. Furthermore, their expression profiles under iron (Fe), aluminum (Al), zinc (Zn), and copper (Cu) stress were evaluated using RT-qPCR. The results demonstrated that most *IbABC* genes exhibited significant differential expression under HM stress. Notably, *IbABCC14* was significantly upregulated under all four metal treatments (Fe, Al, Zn, and Cu) and showed high homology with phytochelatin transporters in *Arabidopsis thaliana* and *Oryza sativa*. This suggests that *IbABCC14* may participate in HM detoxification by mediating vacuolar sequestration. By elucidating the expression patterns of the *IbABC* family under HM stress, this study identifies key candidate genes for breeding sweet potato varieties with enhanced HM tolerance.

## 1 Introduction

Heavy metal (HM) contamination in agricultural soils has become a critical issue due to accelerating industrial development and expanding anthropogenic activities (Chen et al., 2023b; Yu et al., 2023). HMs can undergo long-distance transport and deposit into soil and water, thereby impairing plant growth, yield, and quality; notably, the foliar application of putrescine (Put) on cotton can alleviate chromium toxicity, helping to enhance yields and reduce HM accumulation, which offers a potential strategy for improving plant resilience in contaminated environments (Ghorbani et al., 2025). Cadmium stress inhibits plant growth and induces oxidative damage, while exogenous sulfur (S) application improves the tolerance of Chinese cabbage seedlings to cadmium stress (Lou et al., 2017). In addition to external treatments, plants have evolved diverse defense mechanisms that maintain intracellular ionic balance, reduce toxicity, and improve tolerance to HM stress, contributing to their survival in contaminated environments (Xie et al., 2022; Wang et al., 2026). Among these mechanisms, membrane transport proteins, particularly those in the *ABC* gene family, play a crucial role by facilitating intracellular compartmentalization, vacuolar sequestration, efflux, and homeostatic regulation of HM ions, thereby contributing significantly to HM stress tolerance in sweet potato (Li et al., 2015). Therefore, understanding the transport protein genes responsible for HM transport is essential for enhancing crop tolerance to metal stress and promoting the restoration of contaminated soils (Li et al., 2024; Mujahid and Muhammad, 2024).

The ATP-binding cassette (*ABC*) gene family comprises an ancient and widely distributed class of ATP-dependent transporters involved in the ATP hydrolysis-driven transmembrane transport of diverse substrates (Shen and Li, 2023). These *ABC* transporters facilitate the uptake, intracellular trafficking, and detoxification of endogenous metabolites and HMs, including zinc, manganese, and cadmium, while regulating plant development and responses to abiotic stresses (Zhang et al., 2018; Yang et al., 2025). *ABC* transporters contain conserved nucleotide-binding domains (NBD) and transmembrane domains (TMD). Comparative analysis reveals three classes: Class I, fused NBD and TMD export proteins in Eukaryotes; Class II, non-transport *ABC* proteins without TMDs in both Prokaryotes and Eukaryotes; and Class III, separate NBD and TMD polypeptides forming import proteins in Prokaryotes, with some bacterial export proteins included (Mishra et al., 2019), Comparative sequence analyses categorize *ABC* transporters into full-length transporters, half-transporters, and soluble *ABC* proteins. The family exhibits a high degree of diversity in its structural organization, The full length form consists of two transmembrane domains (TMDs) and two nucleotide binding domains (NBDs), each of which possesses distinct characteristics (Álvarez-Fernández et al., 2025), Half transporters consist of a single transmembrane domain (TMD) and one nucleotide-binding domain (NBD), whereas soluble types retain only a single domain either the NBD or the TMD. Functionally, the NBD provides the energy required for active transport by catalyzing ATP hydrolysis, while the TMD is primarily responsible for substrate recognition, binding, and subsequent redistribution (Chen et al., 2025).

*ABC* transporters play a pivotal role in plant responses to HM stress, a function closely linked to their unique structural and functional characteristics. As a class of transporters that directly drive substrate translocation across membranes via ATP hydrolysis, *ABC* proteins can transport HM ions and their chelates against concentration gradients, either into vacuoles for compartmentalization or out to the extracellular space, thereby achieving intracellular detoxification (Vitelli et al., 2024). While the conserved nucleotide-binding domains (NBDs) are responsible for ATP binding and hydrolysis to provide energy, the highly diverse transmembrane domains (TMDs) empower different members with the ability to recognize various HM-chelate complexes (Kim et al., 2010a). This structural hallmark, consisting of conserved nucleotide-binding domains coupled with diverse transmembrane domains, underpins the functional diversification of the *ABC* transporter family and facilitates their roles in adaptation to HM stress.

Previous studies in various plant species highlight the critical role of *ABC* transporters in HM tolerance. In *Arabidopsis thaliana*, *AtMRP1* (*AtABCC1*) enhances arsenic tolerance when expressed in yeast (Raichaudhuri, 2016). In Arabidopsis, *ABCG36* and *ABCG37* are key to root physiology and stress response. In addition, *ABCG36* helps with pathogen resistance, cadmium efflux, and transport of the auxin precursor IBA. Also, *ABCG37*, involved in IBA, auxin analogs, and coumarin transport, is crucial for iron deficiency responses. Loss of *ABCG37* impairs coumarin secretion and iron uptake, demonstrating the multifunctionality of these proteins (Ziegler et al., 2017). In rice, *ABC* transporter *OsPDR7*, expressed in the exodermis and xylem of roots, is crucial for seed setting and grain weight. Its knockout reduces seed setting and increases zinc accumulation. Yeast expression shows *OsPDR7* zinc efflux activity and regulation of zinc transporter *OsZIP9*, maintaining cellular zinc homeostasis (Lu et al., 2023). In wheat, *TaABCC13*, a homolog of maize LPA1, regulates the glutathione mediated detoxification pathway (Bhati et al., 2015). Silencing *HvABCB25* in barley reduces aluminum tolerance, stunting root growth and increasing aluminum accumulation. Overexpression of *HvABCB25* enhances root growth and dry weight under aluminum stress, indicating its positive role in aluminum tolerance (Liu et al., 2021). Heterologous overexpression of *PtoABCG36* from *Populus tomentosa* and *FvABCC11* from *Fragaria vesca* in *Arabidopsis* reduces cadmium content in shoots and roots, while improving growth and fresh weight under cadmium stress, indicating their role in cadmium tolerance (Wang et al., 2019; Shi et al., 2020). Moreover, multiple *RgABCC* genes in the roots of *Rehmannia glutinosa* are regulated by cadmium stress. Heterologous expression of *RgABCC1* in yeast enhances cadmium tolerance, indicating its involvement in cadmium transport (Yang et al., 2021). Transcriptomic analysis of *Schima superba* showed differential expression of *ABC* transporter genes at days 1, 5, and 10 of manganese treatment. The promoter regions contained stress-responsive cis-acting elements, emphasizing the regulatory role of this gene family in manganese stress responses (Liaquat et al., 2022). Similarly, transcriptomic analysis of maize *ZmABC* gene family under lead stress identified differential expression of multiple members, including *ZmABC012*, *013*, *015*, *031*, *040*, *043*, *065*, *078*, *080*, *085*, *088*, *102*, *107*, *111*, *130*, and *131* and among others. It suggests a key regulatory role of this gene family in responding to lead stress (Xie et al., 2012). Through genome-wide identification and transcriptomic analysis of *Ligusticum chuanxiong* under Cd stress, multiple differentially expressed *LcABC* genes were screened; among these, *LcABCG8*, *LcABCG48*, and *LcABCG108* were identified as key candidate genes involved in the Cd stress response. These findings provide a theoretical foundation for elucidating the underlying molecular mechanisms and developing strategies to reduce cadmium accumulation in this species (Zhen et al., 2025).

Sweet potato (*Ipomoea batatas* (L.) Lam.) is a globally significant food crop renowned for its rich nutritional value and robust environmental plasticity, which enables it to thrive under various adverse climatic and soil conditions (Lyu et al., 2021; Zhao et al., 2024). Due to its high tolerance for poor soil and drought, sweet potatoes are frequently cultivated on marginal lands that often face an elevated risk of HM contamination. The root system serves as the primary organ for water and nutrient uptake, as well as the main entry point for HMs into the plant (Kim et al., 2010b). Unlike seed propagated crops, sweet potato is vegetatively propagated and possesses a high degree of genetic homogeneity, making it an ideal model for studying the molecular mechanisms of HM stress response. Importantly, sweet potato is primarily consumed for its underground storage roots; therefore, the accumulation of HMs directly impacts food safety and human health (Kumar et al., 2022a). Therefore, elucidating the molecular mechanisms of sweet potato response to HM stress specifically the identification of key resistance genes holds significant theoretical value and practical potential. While 129 and 121 ABC transporter genes have been identified and characterized in the genomes of *Arabidopsis* (Sánchez-Fernández et al., 2001) and rice (Garcia et al., 2004), respectively, a systematic report on the number, structural characteristics, and expression patterns of the *ABC* gene family in sweet potato (*IbABC*) under HM stress remains unavailable. To address this gap, this study provides the first genome wide identification and systematic analysis of the *ABC* gene family in sweet potato, comprehensively characterizing their physicochemical properties, protein structures, phylogenetic relationships, collinearity, and promoter cis-acting elements. Furthermore, we utilized RNA sequencing and qRT-PCR to investigate the expression profiles of *IbABC* genes across different organs and under various HM stresses, Fe, Al, Zn, and Cu. These findings not only offer a theoretical foundation for understanding the differentiation and functional evolution of the *IbABC* family but also provide essential insights for breeding sweet potato varieties with enhanced tolerance to HM stress.

## 2 Materials and methods

### 2.1 Identification and physicochemical properties analysis of IbABC gene family members

The identification of *ABC* transporter genes was performed using the *Ipomoea batatas* reference genome assembly (version: *Ipomoea batatas*_pasi3.fa) downloaded from the Sweet potato Genomics Database (https://sweetpotato.com/download_genome.html), while *Arabidopsis ABC* protein sequences were obtained from TAIR (https://www.arabidopsis.org/) (Rhee, 2003).

Using the protein sequences of *Arabidopsis thaliana ABC* transporters as queries, a preliminary search was conducted against the *Ipomoea batatas* genome using the BLASTP program within TBtools-II (v2.222); subsequently, the HMMER program was employed to further screen and identify candidate proteins (Potter et al., 2018), with an E-value cutoff of E < 1×10^−5^ against the *ABC* domain HMM file (PF00005) (El-Gebali et al., 2019) downloaded from the Pfam entry (https://www.ebi.ac.uk/interpro/entry/pfam/#table) in the InterPro database (https://www.ebi.ac.uk/interpro/), to identify candidate proteins containing the *ABC* conserved domain.

To ensure the accuracy of the identification results, the candidate proteins obtained from the screening were submitted to SMART (http://smart.embl-heidelberg.de/) (Marchler-Bauer et al., 2015), NCBI-CDD (https://www.ncbi.nlm.nih.gov/cdd/) (Letunic et al., 2021), and MEME (https://meme-suite.org/meme/tools/meme) for final confirmation and validation of the *ABC* conserved domain. Only those candidate proteins that were identified as containing a complete *ABC* conserved domain by each of the aforementioned tools were included in the final member list.

The physicochemical properties of the final *IbABC* proteins, including amino acid number, molecular weight, isoelectric point, and hydrophilicity, were analyzed using the ExPASy online tool (https://web.expasy.org/protparam) (Gasteiger, 2003). Their subcellular localization was predicted using WoLF PSORT (https://wolfpsort.hgc.jp/) and Cell-PLoc 2.0 (http://www.csbio.sjtu.edu.cn/bioinf/Cell-PLoc-2/) (Chou and Shen, 2010).

### 2.2 Chromosomal localization and phylogenetic analysis of IbABC gene family members

The GFF3 annotation file of the sweet potato genome was downloaded from the sweet potato genome database (https://sweetpotato.com/download_genome.html) to obtain the chromosomal positions of *IbABC* genes, the lengths of each chromosome, and the gene structures (Bailey et al., 2006), Subsequently, TBtools-II (version v2.222) was employed to visualize the chromosomal gene density and the physical locations of *IbABC* genes (Hu et al., 2023). A phylogenetic tree of *IbABC* and *AtABC* proteins was constructed using the Neighbor-Joining (NJ) method in MEGA11 software with 1,000 bootstrap replicates, and the resulting tree was further visualized using the online tool EvolView v2 (https://www.evolgenius.info/evolview-v2/#login) (Chen et al., 2024).

### 2.3 Gene structure and conserved motifs of IbABC gene family members

Exon/intron information for sweet potato *ABC* genes was extracted from the sweet potato gene annotation file. Subsequently, conserved regions of the *IbABC* proteins were analyzed using online websites and TBtools software, with the number of motifs set to 10 and the motif E-value threshold set to E<1×10−10 (Chen et al., 2023a). Simultaneously, the online MEME tool (https://meme-suite.org/meme/) was used to analyze the conserved structures of the *IbABC* family members.

### 2.4 Collinearity of IbABC genes and Ka/Ks analysis

The genome data and gene annotation files for *Arabidopsis thaliana* and *Oryza sativa* were downloaded from The *Arabidopsis thaliana* Information Resource (TAIR, https://www.arabidopsis.org/), and Phytozome database (https://phytozome-next.jgi.doe.gov/). Gene duplication events within the sweet potato *ABC* family were analyzed using MCScanX. Collinearity analysis was performed between the sweet potato *ABC* family genes and the genes from two closely related species, as well as *Arabidopsis thaliana*, *Oryza sativa*, *Manihot esculenta*, and *Smallanthus sonchifolius* (Wang et al., 2012). DupGen_finder (https://github.com/qiao-xin/DupGen_finder) was used to identify various types of gene duplication events (Qiao et al., 2019). non-synonymous substitution rate (Ka), synonymous substitution rate (Ks), and Ka/Ks ratio for duplicate gene pairs were calculated using KaKs_Calculator 2.0 (Wang et al., 2010).

### 2.5 Analysis of the promoter cis-regulatory elements of the IbABC gene

The 2000 bp upstream region of the *IbABC* gene was extracted from the sweet potato genome to serve as the promoter sequence. Putative cis-acting elements were predicted using the online program PlantCARE (http://bioinformatics.psb.ugent.be/webtools/plantcare/html/), and the results were visualized using TBtools (Lescot et al., 2002).

### 2.6 Transcriptome data analysis of IbABC gene family expression in different organs

Public transcriptome sequencing data for eight different tissues of the sweet potato cultivars “Yan 252” were retrieved from the China National Center for Bioinformation (CNCB https://ngdc.cncb.ac.cn, accession number CRA000606) (Ding et al., 2017). Additionally, transcriptome sequencing was performed by our research team on the fibrous roots, initial tuberous roots, and expanding tuberous roots of the “Annayu” cultivar (GSA project number PRJCA036206). Using HISAT2, the clean reads were aligned to the reference genome, and the mapped reads were quantified with featureCounts. Subsequently, based on the log2(FPKM + 1) values, the TBtools software was used to visualize the gene expression levels in a heatmap, in order to analyze the tissue-specific expression patterns of the *IbABC* genes in the two varieties. Expression profiles of *IbABC* genes in different tissues were extracted from the transcriptome data of the “Yan 252” cultivar, with cartoon schematic diagrams generated using Adobe Illustrator, and expression heatmaps of representative genes generated using TBtools software (Wang et al., 2021).

### 2.7 Experimental materials

In this experiment, a commercially preformulated modified basal Hoagland nutrient solution was used. HM treatments were applied by adding analytical-grade reagents to this basal solution: Cu as copper (II) sulfate pentahydrate, Al as aluminum chloride hexahydrate, Fe as sodium ferric ethylenediaminetetraacetate (to ensure stability and bioavailability), and Zn as zinc sulfate heptahydrate. The pH of the Al treatment solution and the control solution was uniformly adjusted and maintained at 4.5 ± 0.1 (Khan et al., 2024), while the pH of the other treatment solutions and control solutions was uniformly adjusted and maintained at 5.5 ± 0.1 (Kumar et al., 2022b; Shi et al., 2023), All solutions were freshly prepared before each application.

The selected concentrations were based on ranges reported in previous studies and preliminary experiments, with the aim of simulating sub-lethal stress conditions to investigate plant physiological response mechanisms rather than inducing acute toxicity.The applied concentrations—10 mg/L Cu, 50 mg/L Al, 50 mg/L Fe, and 50 mg/L Zn (He et al., 2021; Tian et al., 2021; Gao et al., 2025) were sufficient to induce stress-related phenotypes within the experimental period and were appropriate for comparing the toxicity profiles and plant tolerance across different metals.

### 2.8 Plant growth and HM treatment

The pot experiment was conducted using the sweet potato variety ‘Xinxiang’ at the Sanya Nanfan Research Institute experimental base of Hainan University. Uniform sweet potato vine cuttings (25 cm in length) were prepared by cutting the shoots from the plants, while retaining the top three fully expanded leaves. The vine cuttings were planted at an oblique angle in plastic pots (3-L capacity) equipped with drainage trays. Each pot measured 16 cm in top diameter, 12 cm in bottom diameter, and 17 cm in height. The optimal planting depth was standardized by inserting three nodes of each cutting into the quartz sand. The quartz sand substrate was prepared by mixing three particle-size fractions (0.2–0.4 mm, 0.4–0.6 mm, and 0.8–1.2 mm) in a 1:2:1 ratio, To maximize the controllability of the plant rhizosphere environment and minimize complex and uncontrollable metal adsorption and speciation transformation caused by soil organic matter and clay minerals.

At 0, 3, 6, 12, 24, and 48 hours after each treatment, fibrous root samples were collected from three independent biological replicates of sweet potato, immediately flash-frozen in liquid nitrogen, and stored at –80 °C. All metals were applied as single stress factors to separate experimental groups. For each metal treatment group and its corresponding sampling time point, process-matched parallel control groups were established, Control plants were cultivated and sampled simultaneously with the treatment groups, ensuring that data from each time point could be directly compared with its time-matched control. After 10 days of continuous stress treatment, the roots of three plants from each treatment group were selected, rinsed with distilled water, and gently dried. All root samples were scanned using an EPSON GT-X980 scanner, and the scanned images were analyzed using RhizoVision Explorer software to obtain root architecture parameters (Wang et al., 2021) (**Table 1**).

**Table 1:** Parameters for iron, aluminum, zinc, and copper stress treatments.

	Chemical Form (Reagent)	Treatment pH	Concentration (mg/L)	Concentration (µM)
Cu	CuSO₄·5H₂O	5.5 ± 0.1	10	40.1 (Cu²⁺)
Al	AlCl₃·6H₂O	4.5 ± 0.1	50	207 (Al³⁺)
Fe	NaFe-EDTA	5.5 ± 0.1	50	119 (Fe³⁺)
Zn	ZnSO₄·7H₂O	5.5 ± 0.1	50	174 (Zn²⁺)

### 2.9 RNA extraction and real-time quantitative RT-PCR analysis

Total RNA was extracted from sweet potato fibrous root samples collected at 0, 6, 12, 24, 48 h, and 10 d after stress treatment using the RNAprep Pure Plant Kit (Tiangen, Beijing, China, DP437). The concentration and quality of the extracted RNA were assessed via spectrophotometry, and the purified RNA was subsequently reverse-transcribed into cDNA using the HiScript II Q RT SuperMix (Vazyme, Nanjing, China, R223). RT-qPCR analysis of *IbABCs* was performed on a qTOWER 3G Real-Time PCR System (Analytik Jena, Germany) with 2× Q3 SYBR qPCR Master Mix (ToloBio, China), using β-actin as the internal reference gene. Specific primers were designed using Primer Premier 5.0 (sequences listed in **Supplementary Table 2**), and relative expression levels were calculated using the 2^–ΔΔCT method across three biological replicates for all samples.

### 2.10 Homology comparison and analysis of IbABCC14

To further validate the function of the candidate gene, we identified homologs of *IbABCC14* in the model crops *Arabidopsis thaliana* and *Oryza sativa* using the “Find Best Homology” tool in TBtools. The analytical results were visualized via TBtools, while correlation plots were generated using Origin 2024 and multiple sequence alignments were performed using DNAMAN 9 software.

### 2.11 Statistical analysis

Statistical analysis was performed using Microsoft Excel 2020, with mean differences between treatments evaluated via the Least Significant Difference (LSD) test in Statistix 9; all data are presented as the means of three replicates, where asterisks denote levels of significance (*P < 0.05, **P < 0.01, ***P < 0.001) and different lowercase letters indicate significant differences (*P* < 0.05), while bar charts and correlation heatmaps were generated using GraphPad Prism 8 and Origin 2024 (Wang et al., 2025).

## 3 Results

### 3.1 Identification and functional analysis of IbABC gene family members

**Figure 1:**
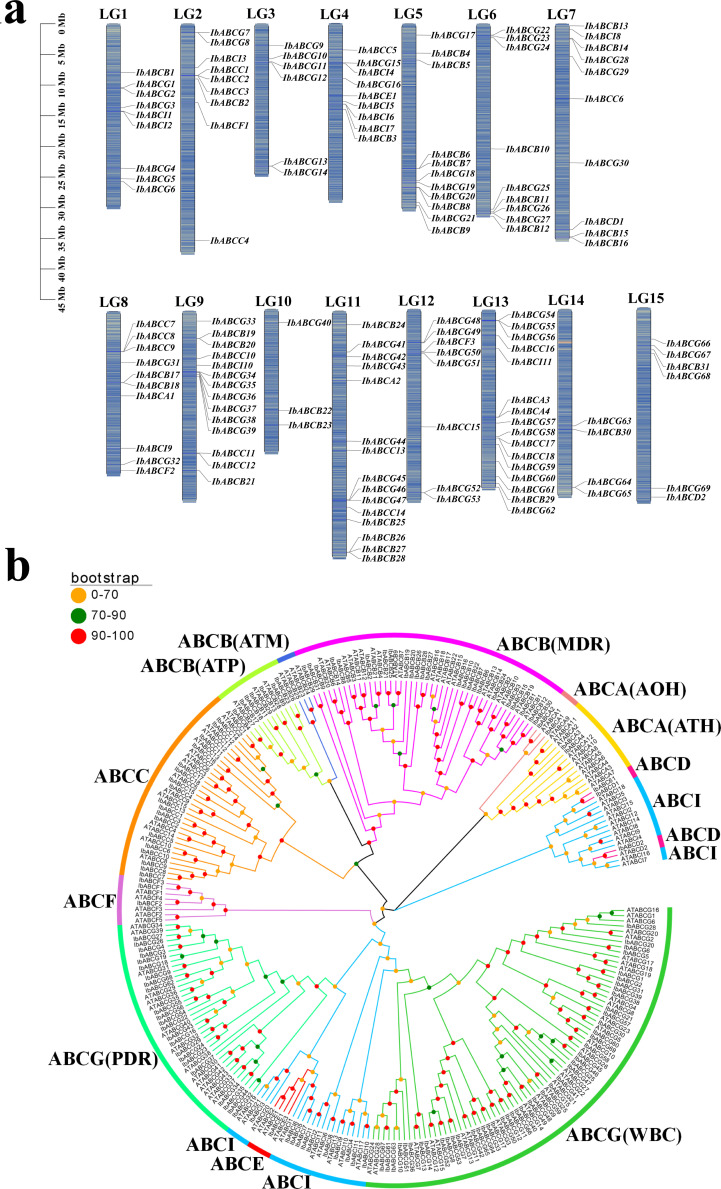
Chromosomal localization and phylogenetic tree of the *IbABC* gene family. **(a)** Chromosomal localization and distribution of *ABC* genes in sweet potato (*Ipomoea batatas*). **(b)** An unrooted phylogenetic tree of the *ABC* protein family from sweet potato and Arabidopsis thaliana, where different colors represent distinct subfamilies.

A total of 139 *ABC* genes were identified from the sweet potato genome based on the presence of characteristic domains. After removing redundant sequences, these genes were classified into eight subfamilies. Based on their chromosomal locations, the genes were designated as *IbABCA1*–*IbABCA4*, *IbABCB1*–*IbABCB31*, *IbABCC1*–*IbABCC18*, *IbABCD1*–*IbABCD2*, *IbABCE1*, *IbABCF1*–*IbABCF3*, *IbABCG1*–*IbABCG69*, and *IbABCI1*–*IbABCI11*. The *IbABC* genes were unevenly distributed across the 15 chromosomes. Chromosome 13 contained the highest number of genes (16 genes, 11.51%), whereas chromosome 10 had the fewest (3 genes, 2.16%) (**Figure 1a**). This uneven distribution suggests that certain chromosomes may play specific roles in the regulation of *ABC* genes, but this hypothesis requires further experimental validation. The uneven distribution could also reflect evolutionary events like gene duplication or transposition.

The physicochemical properties of the *IbABC* family members varied significantly (**Supplementary Table 1**). The protein length ranged from 203 amino acids (*IbABCI6*) to 2397 amino acids (*IbABCB29*), with corresponding molecular weights from 22.76 kDa (*IbABCI6*) to 264.48 kDa (*IbABCB29*). The theoretical isoelectric points (pI) spanned from 5.57 (*IbABCF2*) to 9.83 (*IbABCI5* and *IbABCI7*), with 101 proteins being acidic and 38 proteins being basic. The instability index varied between 26.49 (*IbABCI10*) and 56.13 (*IbABCG57*), suggesting that 65 proteins are stable and 74 are unstable. Furhtermore, the GRAVY values indicated that 92 *IbABCs* are hydrophobic, while 47 are hydrophilic. Additionally, Subcellular localization predictions showed that 83.45% of the *IbABC* family members (116 proteins) are in the plasma membrane, suggesting their primary functional role there. Notably, none of the members from the *IbABC* I subfamily are in the plasma membrane, implying that this subfamily may play distinct roles within the plant.

To further investigate the evolutionary relationships of the *IbABC* family, a Neighbor-Joining (NJ) phylogenetic tree was constructed using the protein sequences of the 139 *IbABC* and 129 *AtABC* members from *Arabidopsis thaliana* (MEGA-X software) (**Figure 1b**). The phylogenetic analysis showed that all sequences were classified into eight subfamilies (*ABCA*–*ABCG* and *ABCI*), with each group containing multiple genes from the two species. This finding was aligned with classification studies conducted in *Arabidopsis thaliana*. Among 139 *IbABC* proteins, the *ABCG* subfamily was the largest, comprising 48.92% of the total *IbABC* genes, and was further divided into two evolutionary branches: (i) PDR group (22 members) and (ii) WBC group (46 members). The *ABCB* subfamily was the second largest, containing 31 members (22.30%), while the *ABCE* subfamily had only one member. Genes located on adjacent branches showed closer evolutionary relationships, suggesting they may perform similar biological functions. These evolutionary patterns provide valuable insights into the functional diversification of the *IbABC* gene family in sweet potato. The distinct separation of subfamilies also hints at specialized roles that each group may play in the plant’s response to various stresses or physiological needs, offering a foundation for further functional studies.

### 3.2 Analysis of IbABC gene structure and conserved motifs

Conserved protein motifs are crucial for understanding the function of proteins. In the *IbABC* amino acid sequences, ten conserved motifs were detected, with individual genes containing 8–10 motifs. Motifs 1, 2, 3, 6, 7, 8, and 10 were present in all *IbABC* proteins, highlighting their functional importance across the entire gene family. Members within the same subfamily exhibited highly similar motif compositions. However, clear differences were observed between the *IbABCG* (PDR) and *IbABCG* (WBC) subfamilies, with motifs 9 and 10 being specifically present in *IbABCG* (PDR) and *IbABCG* (WBC), These differences suggest functional differentiation among subfamilies, however, further experimental validation is required.

**Figure 2:**
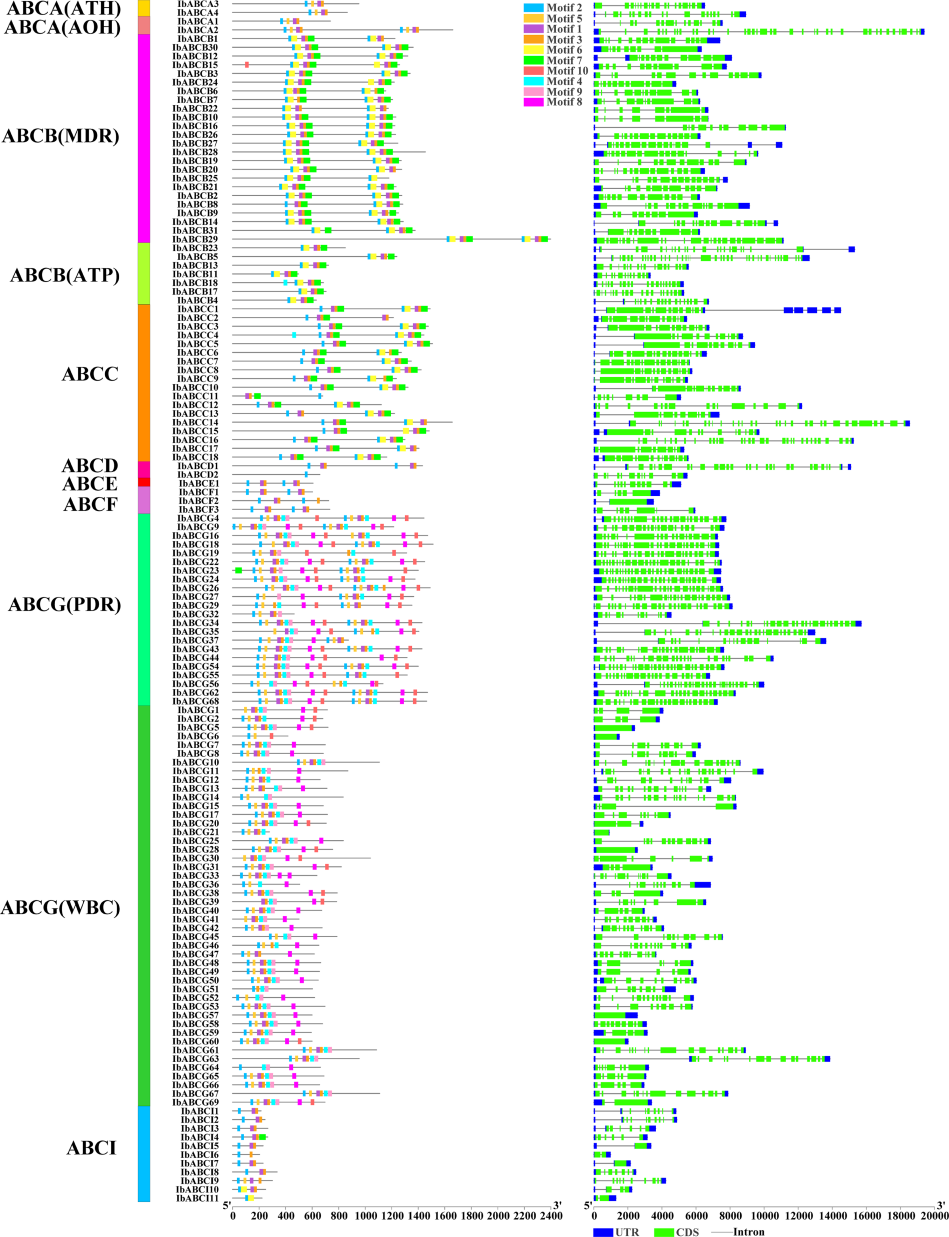
Analysis of gene structures, conserved domains, and motifs of *ABC* proteins in sweet potato. The structural components of the *ABC* genes—including untranslated regions (UTRs), coding sequences (CDS), and introns—are illustrated, with blue boxes representing UTRs, green boxes representing CDS, and thin black lines representing introns. Additionally, conserved motifs within the *ABC* proteins were identified using the MEME program, where ten distinct motifs (numbered 1–10) are displayed in various colors to highlight their distribution and composition.

The exon-intron structures of the *IbABCG* (PDR) and *IbABCG* (WBC) subfamilies were significantly different. In the *IbABCG* (WBC) group, genes like *IbABCG5*, *IbABCG6*, *IbABCG21*, *IbABCG57*, and *IbABCG60* have a minimal intron configuration, with three exons and no introns, whereas *IbABCG69* contains four exons and one intron. On the other hand, genes in the *IbABCG* (PDR) subfamily contain more exons and introns, indicating a higher potential for alternative splicing. Within the *ABCC* subfamily, *IbABCC14* stands out with the most complex gene structure, containing 34 exons and 31 introns, which may reflect its broad functional role compared to other *ABCC* members (**Figure 2**). These findings suggest that the structural complexity of the *IbABC* gene family may be closely related to their functional diversification. The variation in exon-intron structures, especially within the *IbABCG* (PDR) and *IbABCG* (WBC) subfamilies, may provide insights into the evolutionary adaptations of the *ABC* transporters in response to environmental stresses or metabolic needs in sweet potato. Additionally, the conserved motifs across the gene family are likely to play critical roles in substrate recognition, transport efficiency, and functional specialization, which will be important for further functional characterization of these genes.

### 3.3 Collinearity analysis of the IbABC gene family and the ratio of non-synonymous substitutions (Ka) to synonymous substitutions (Ks)

**Figure 3:**
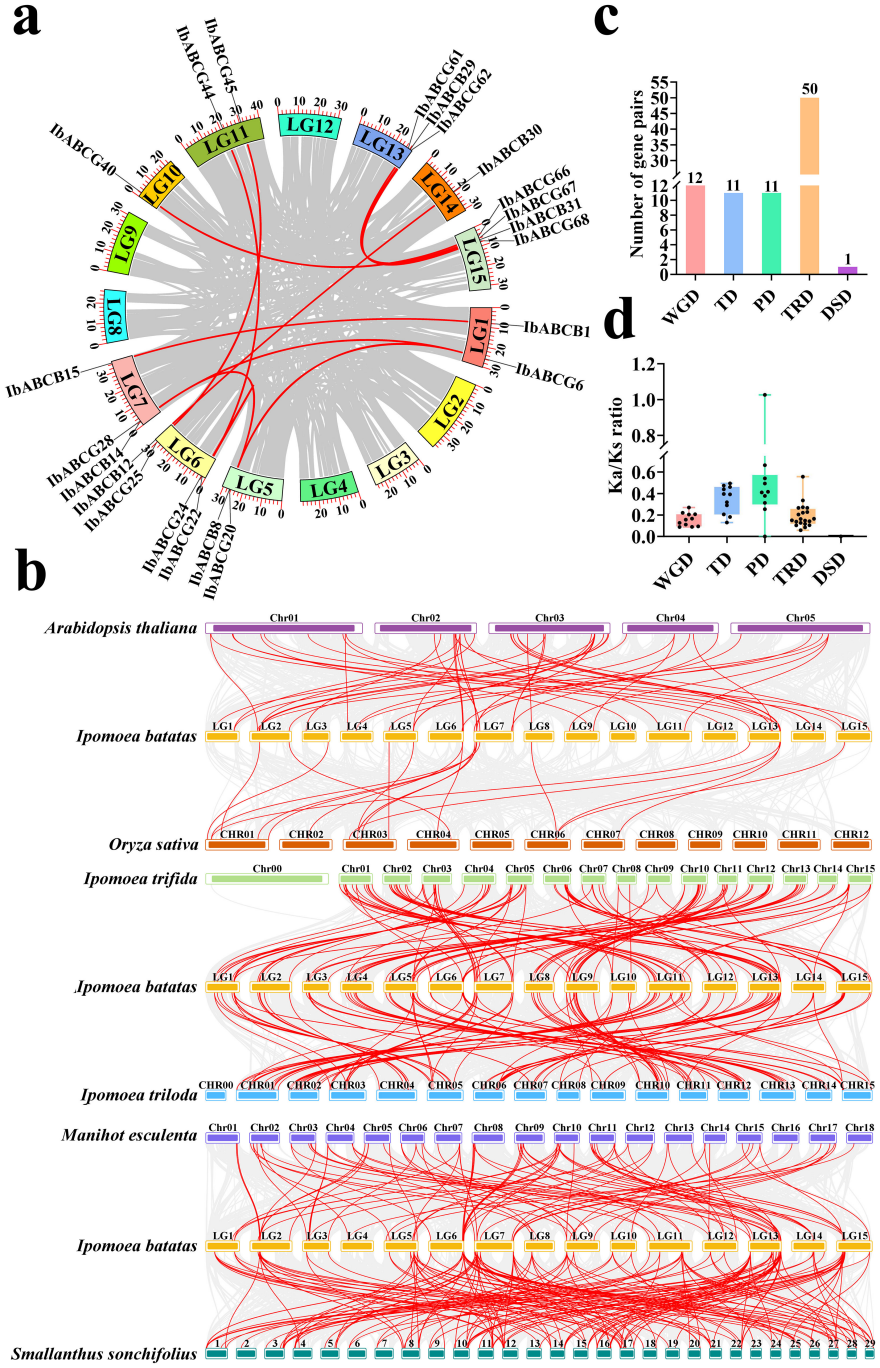
Collinearity analysis and replication mode Ka/Ks of the *IbABC* gene family. **(a)** Intragenomic synteny of *IbABC* genes; **(b)** Interspecific synteny between *Ipomoea batatas* and *Arabidopsis thaliana, Oryza sativa, Ipomoea triloba, Ipomoea trifida, Manihot esculenta*, and *Smallanthus sonchifolius*; **(c)** Gene pairs derived from different duplication modes in the *IbABC* gene family, including whole-genome duplication (WGD), transposed duplication (TRD), dispersed duplication (DSD), tandem duplication (TD), and proximal duplication (PD); **(d)** A scatter plot of Ka/Ks analysis.

Gene duplication plays a crucial role in plant evolution, contributing significantly to the expansion of gene families. To investigate potential relationships and duplication events within the *IbABC* family, collinearity analysis was performed using BLASTp and MCScanX. The intraspecific analysis identified 12 segmental duplication events among *IbABC* members, including *IbABCG6*/*IbABCG20*, *IbABCB8*/*IbABCB14*, *IbABCG44*/*IbABCG22*, *IbABCG22*/*IbABCG24*, *IbABCG6*/*IbABCG28*, *IbABCB1*/*IbABCB15*, *IbABCG40*/*IbABCG66*, *IbABCG45*/*IbABCG25*, *IbABCG61*/*IbABCG67*, *IbABCG62*/*IbABCG68*, *IbABCB30*/*IbABCB12*, and *IbABCB29*/*IbABCB31* (**Figure 3a**). Further, interspecific collinearity was assessed by comparing six species *Arabidopsis thaliana*, rice, *Ipomoea triloba*, *Ipomoea trifida*, cassava, and *Smallanthus sonchifolius*. This analysis identified 606 homologous gene pairs (**Figure 3b**). The highest number of collinear pairs occurred between sweet potato and *S. sonchifolius* (177 pairs), followed by *I. triloba* and *I. trifida* (both with 131 pairs), *cassava* (95 pairs), and *A. thaliana* (53 pairs). Rice, in contrast, exhibited the fewest collinear pairs (19 pairs), indicating a closer evolutionary relationship between sweet potato and dicot species than with monocots.

To further investigate the mechanisms driving the expansion of the *IbABC* gene family, duplication patterns were analyzed. A total of 85 duplicated gene pairs were identified, encompassing five duplication modes, whole-genome duplication (WGD, 12 pairs), tandem duplication (TD, 11 pairs), proximal duplication (PD, 11 pairs), transposed duplication (TRD, 50 pairs), and dispersed duplication (DSD, 1 pair) (**Figure 3c**). These results indicate that TRD is the predominant driver of *IbABC* family expansion. To assess the evolutionary dynamics of the duplicated genes, Ka/Ks ratios (non-synonymous/synonymous substitution rates) were calculated for all duplicated *IbABC* gene pairs in sweet potato. Most of these pairs exhibited Ka/Ks values <1 (**Figure 3d**), suggesting that purifying selection plays a critical role in the evolution of the *IbABC* gene family.

### 3.4 Analysis of cis-regulatory elements in the IbABC gene family

**Figure 4:**
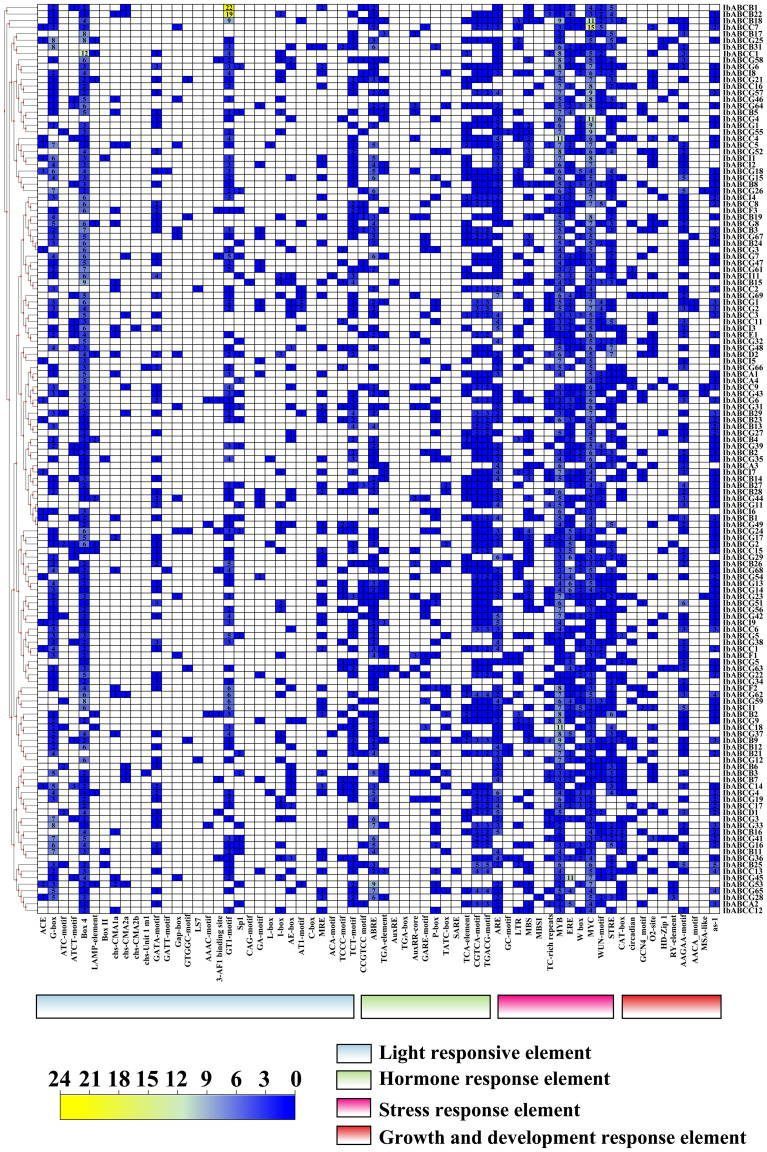
Cis-element analysis of the *IbABC* gene family. Cis-elements are categorized into four groups: Light responsive element, Hormone response element, Stress response element, and growth and development response element, with each category represented by a distinct color.

To understand the functional characteristics of *IbABC* gene family, cis-acting elements in their promoter regions were identified using PlantCARE. A total of 66 cis-element types were detected and classified into four major categories: light responsive, growth and development related, hormone responsive and stress responsive elements. These cis-elements were then quantified and visualized through heatmap profiling (**Figure 4**). The analysis revealed a total 5,353 cis-elements across all *IbABC* genes. Among these, stress responsive elements were the most abundant, making up 2,450 elements, followed by light-responsive elements (1,576) and hormone-responsive elements (808). While growth and development-related elements were the least common, with only 519 elements identified. Within the stress-responsive category, MYB elements were the most abundant, with MYC elements following closely behind. For the light-responsive group, Box 4 was the dominant motif, followed by the G-box. Among hormone-responsive elements, ABREs appeared most frequently, followed by MeJA-responsive motifs (CGTCA- and TGACG-motifs). The growth and development related elements were less frequent, with AAGAA-motif and as-1 being the most represented. Interestingly, MYC elements were absent only in *IbABCB5* and *IbABCF3* within the gene family, although both genes contained a high number of MYB and Box 4 elements. This pattern suggests that MYC elements are relatively conserved across the *IbABC* gene family, while their absence in specific genes may be associated with distinct regulatory functions. Based on these analyses, the *IbABC* gene family may play roles in plant growth and development. Furthermore, the analysis of the 2000 bp upstream promoter sequences of all gene family members, We identified one conserved motif (CAAAT) that is universally present in all members of the *IbABC* family, highlighting a high degree of evolutionary conservation (**Supplementary Figure 1**).

### 3.5 Effects of iron, aluminum, zinc, and copper on the growth and development of sweet potato vine cuttings

**Figure 5:**
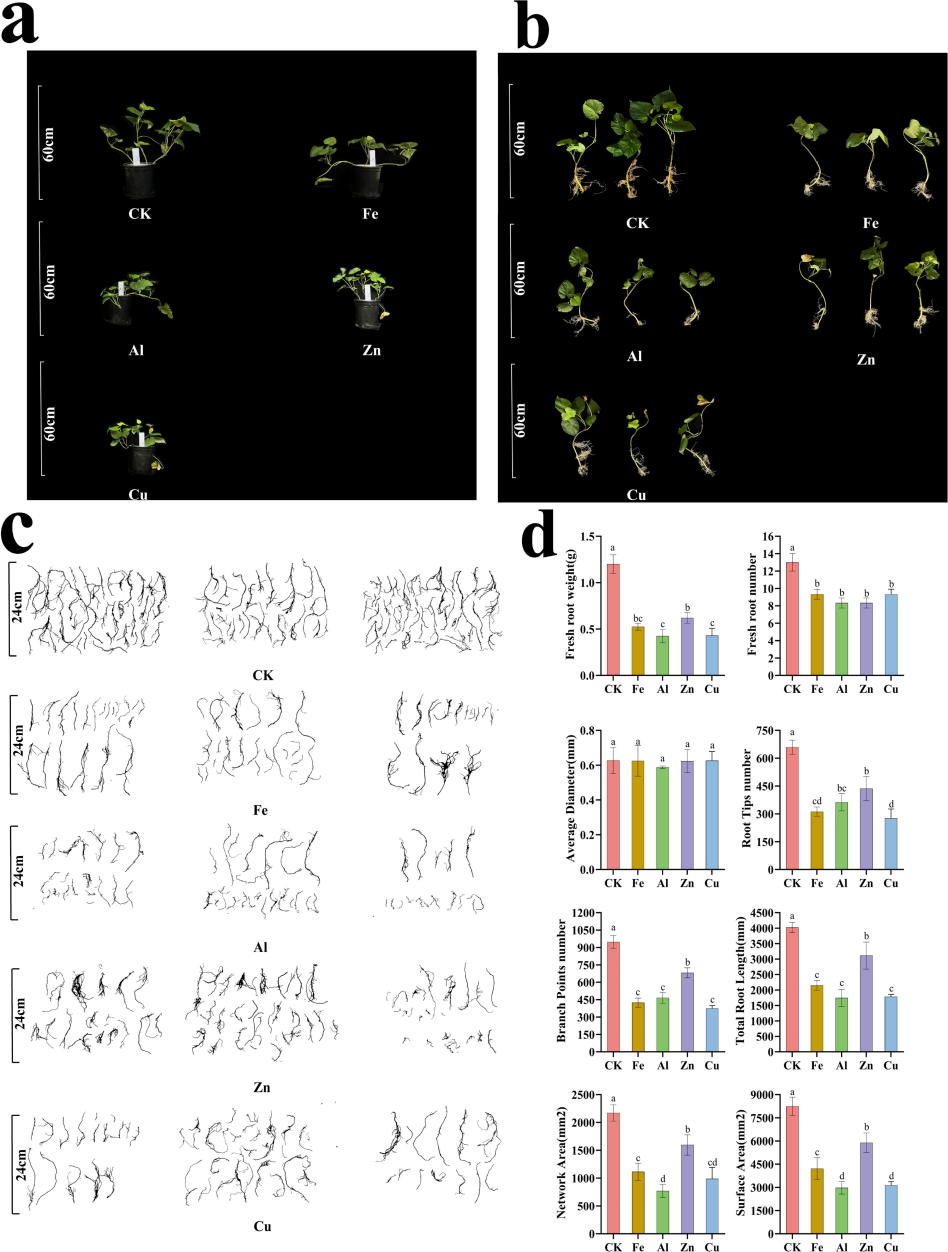
Analysis of sweet potato growth and development under Iron (Fe), Aluminum (Al), Zinc (Zn), and Copper (Cu) treatments. **(a)** Phenotypic changes in sweet potato plants under Fe, Al, Zn, and Cu stress compared to the control (CK). **(b)** Root morphological changes, including root condition under different treatments. **(c)** Root scanning images, showing the root structure and distribution under these stresses. **(d)** Data for fibrous root weight, fibrous root number, average diameter, root tip number, root branching point number, total root length, root network area, and root surface area under Control (CK), Fe, Al, Zn, and Cu treatments. Data are presented as mean ± standard deviation (n=3). Different lowercase letters indicate significant differences (P < 0.05) as determined by one-way ANOVA.

To investigate the effects of Fe, Al, Zn, and Cu stress on the growth and development of sweet potato vine cuttings, plants were exposed to these four elements for 10 days. Morphological observations (**Figures 5a, b**) showed that, compared with the control (CK), the growth vigor of the treated plants, including plant height, leaf condition, and root development, was reduced to varying degrees under Fe, Al, Zn, and Cu stress. Particularly, the plants exhibited stunted growth and poor leaf conditions under these stresses, highlighting the detrimental effects of metal exposure. The roots showed obvious signs of stress, including limited branching, reduced root length, and fewer fine roots, further emphasizing the negative impact of HMs on root architecture.

Root scanning (**Figure 5c**) further revealed significant changes in root structure, distribution, and density under Fe, Al, Zn, and Cu stress. Compared with the control group, all measured root morphological characteristics, except for the average root diameter, were significantly reduced under HM stress (*P* < 0.05) (**Figure 5d**). Specifically, the dry weight of fine roots decreased by approximately 2.28-fold, 2.82-fold, 1.93-fold, and 2.79-fold under Fe, Al, Zn, and Cu stress, respectively, indicating a severe reduction in root biomass. The number of fine roots decreased by approximately 1.39-fold, 1.56-fold, 1.56-fold, and 1.39-fold, respectively, while the number of root tips decreased by approximately 2.11-fold, 1.81-fold, 1.51-fold, and 2.39-fold, respectively. This suggests a reduced ability of roots to absorb water and nutrients, which may impair the overall growth and survival of the plant.

In addition to the changes mentioned above, under metal stress, the number of root branching points decreased by approximately 2.23 times, 2.03 times, 1.39 times, and 2.54 times, respectively. Similarly, the total root length, a key indicator of root development, reduced by approximately 1.87 times, 2.30 times, 1.29 times, and 2.25 times, respectively. The root network area, reflecting the spatial exploration range of the root system, decreased by approximately 1.95 times, 2.82 times, 1.36 times, and 2.20 times under Fe, Al, Zn, and Cu stress, respectively. Similarly, the root surface area, which plays a crucial role in nutrient and water absorption, decreased by approximately 1.96 times, 2.77 times, 1.40 times, and 2.62 times compared to the control group, respectively. These quantitative results highlight the significant impact of HM stress on root growth and structure. The changes in root morphology and structure under iron, aluminum, zinc, and copper stress indicate that these metals severely hinder the plant’s ability to maintain effective root function, thereby limiting the plant’s overall development and productivity.

The results of this experiment indicate that exposure to Fe, Al, Zn, and Cu significantly disrupts root growth and morphology. These disruptions are likely to have a ripple effect on other vital physiological processes, including nutrient uptake, water absorption, and overall plant vigor. This suggests that HM contamination in the soil may pose a significant barrier to sweet potato cultivation, particularly in regions where these metals are present in high concentrations. Additionally, the findings highlight the importance of developing effective strategies to address HM stress, such as soil amendments or breeding stress-resistant varieties.

### 3.6 Analysis of expression patterns of the IbABC gene family

**Figure 6:**
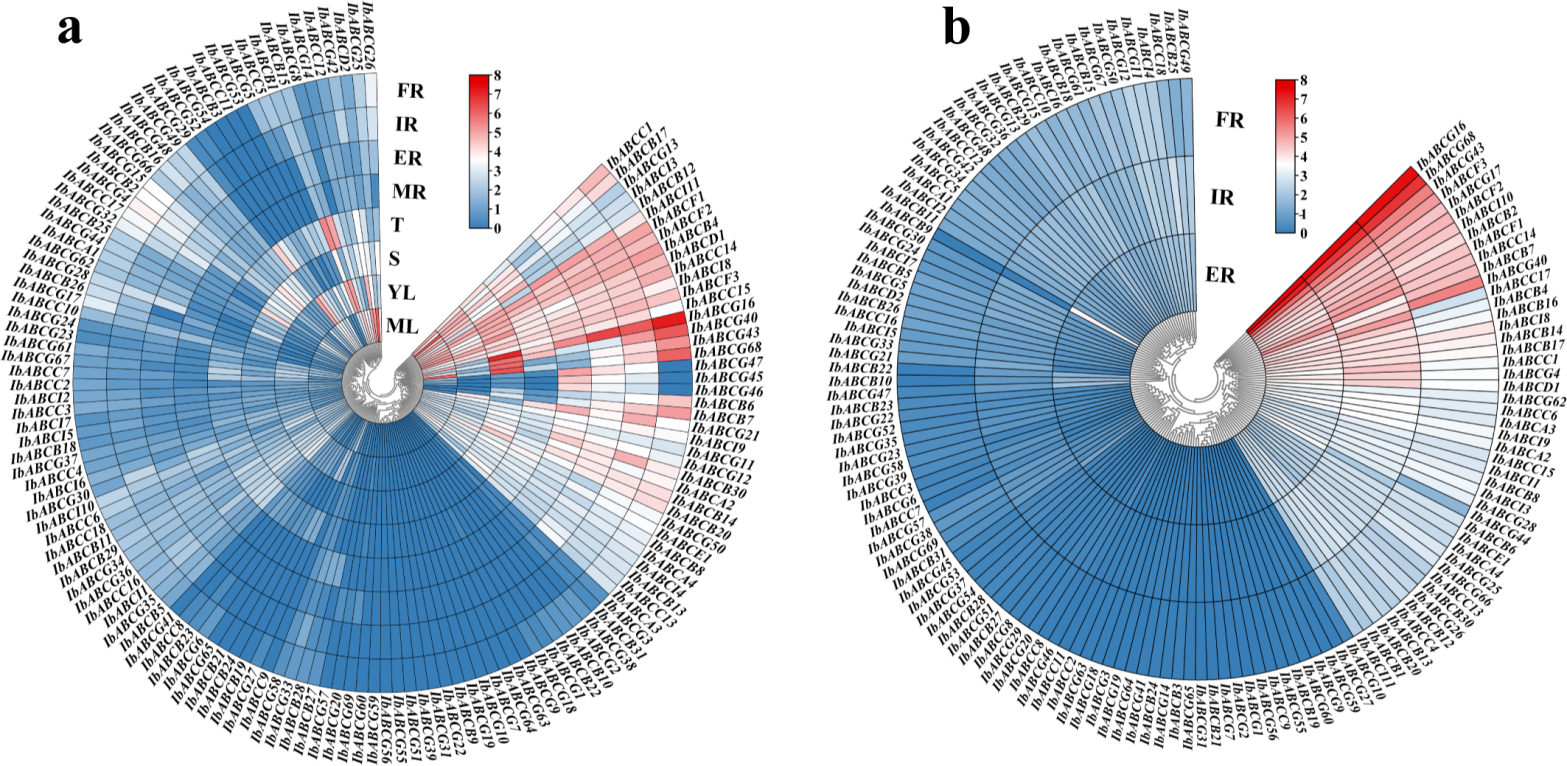
Expression profiles of *IbABC* family genes in different organs of sweet potato. **(a)** Expression profile of sweet potato cultivar ‘Yan 252’. **(b)** Expression profile of sweet potato cultivar “Annayu”. FR, fibrous root; IR, initiating root; ER, expanding root; MR, mature root; T, stem; S, shoot; YL, young leaf; ML, mature leaf. The heatmap was generated using log2 (FPKM + 1) transformed values with row scaling. The color gradient represents relative expression levels.

To systematically characterize the expression patterns of the *IbABC* gene family, RNA-seq transcriptomic data from different tissues of the sweet potato cultivar “Yan 252”-including fibrous root (FR), initial root (IR), expending root (ER), mature root (MR), stem (T), shoot (S), young leave (YL), and mature leave (ML)-were analyzed. The results indicated that these expression profiles reflect a complex gene regulatory network during sweet potato growth and development (**Figure 6a**). Based on their expression characteristics, the *IbABC* gene family can be classified into four groups: Group I genes are highly expressed in all tissues, such as *IbABCF1*/*2*/*3*, *IbABCB4*, *IbABC18*, *IbABD1*, and *IbABCC14*; Group II genes are predominantly expressed in roots, including *IbABCC1* and *IbABCG16*; Group III genes are mainly expressed in shoot, young leaves, and mature leaves, but show low or negligible expression in other tissues, such as *IbABCG8*/*29*/*42*/*52*; and Group IV genes are primarily highly expressed in stems, such as *IbABCG32*. These findings suggest that *IbABC* genes exhibit pronounced tissue-specific regulatory roles during sweet potato development.

Furthermore, we analyzed RNA-seq expression data from fibrous root (FR), initial root (IR), and expanding root (ER) of another sweet potato cultivar, “Annayu” (**Figure 6b**). The results showed that genes such as *IbABCG16*/*17*/*68*/*43*, *IbABCF3*/*2*, and *IbABCC14* display distinct expression patterns among different root types. The high consistency of expression trends across the three root tissues not only validates the reliability of the RNA-seq data but also suggests that these *IbABC* genes may have relatively conserved functions across different cultivars and stages of root development.

**Figure 7:**
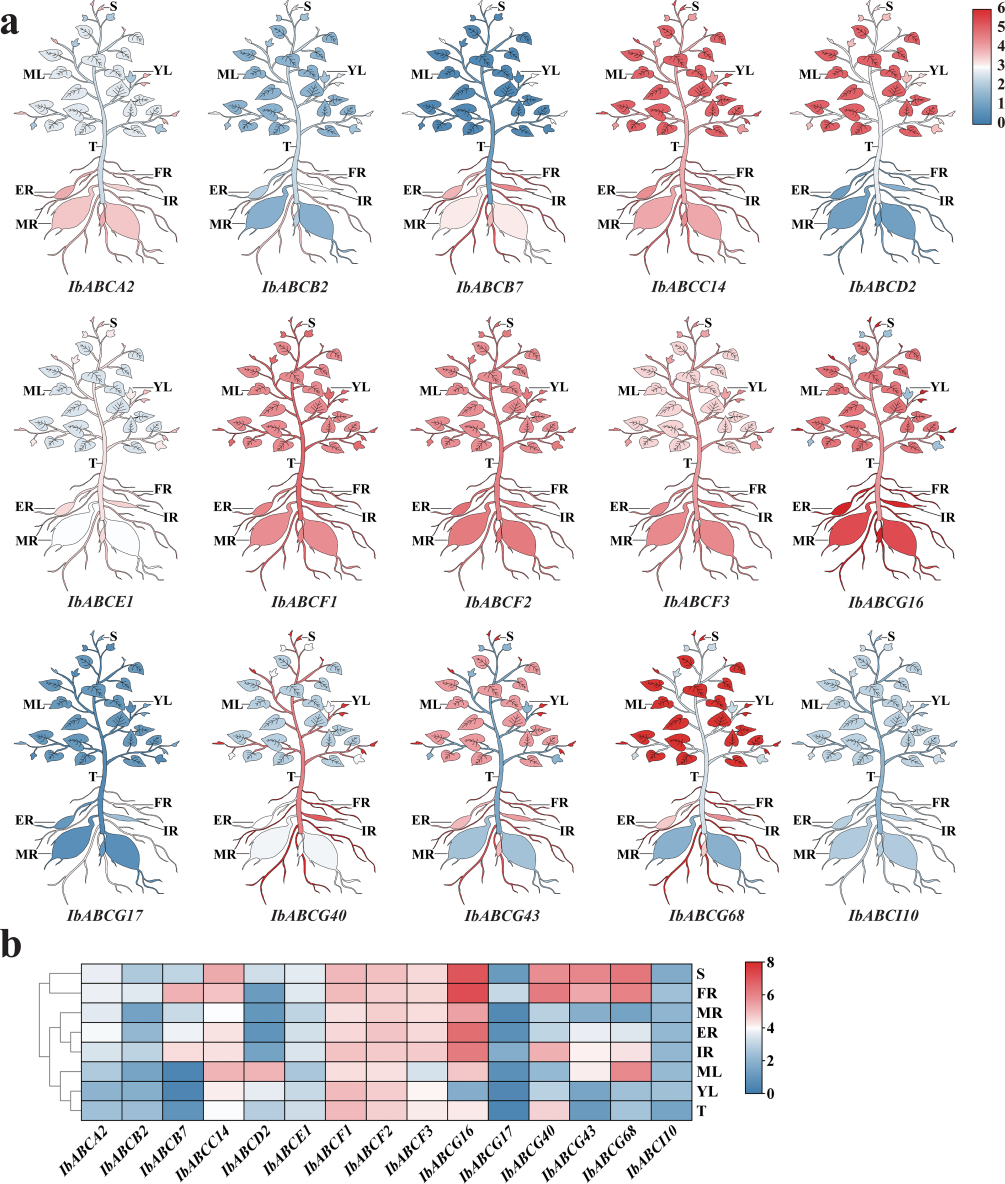
Exquisite heatmaps and integrated group heatmaps illustrating the differential expression of representative *IbABC* genes. **(a)** Detailed heatmaps of representative *IbABC* genes from each branch; FR, fibrous root; IR, initiating root; ER, expanding root; MR, mature root; T, stem; S, shoot; YL, young leaf; ML, mature leaf. **(b)** Integrated group heatmap showcasing the expression profiles of representative *IbABC* genes.

To more intuitively display the expression distribution of *IbABC* family genes in different tissue locations, we selected 1–5 representative genes from each evolutionary branch and drew heatmaps reflecting their expression characteristics (**Figure 7a**). As shown in the figure, *IbABCA1* of the *ABCA* group is mainly expressed in four types of roots and buds; *IbABCB2* and *IbABCB7* of the *ABCB* group show higher expression levels in fibrous roots. *IbABCC14* of the *ABCC* group is actively expressed in all detected tissues. *IbABCD2* of the *ABCD* group is mainly expressed in old leaves. *IbABCE1* of the *ABCE* group has high expression in stems, buds, and four types of roots. *IbABCF1*, *IbABCF2*, and *IbABCF3* of the *ABCF* group show high expression levels in all tissues. In the *ABCG* group, *IbABCG16*, *IbABCG40*, and *IbABCG43* have high expression in buds and fibrous roots; *IbABCG17* is mainly expressed in fibrous roots; whereas *IbABCG68* is highly expressed in fibrous roots, buds, and old leaves. *IbABCI10* of the *ABCI* group has strong expression signals in fibrous roots. In addition, we normalized and integrated the expression data of these 15 genes and drew a global heatmap to compare the expression profiles of representative genes from different branches (**Figure 7b**). In summary, this study indicates that *IbABC* family genes exhibit diverse expression patterns in various sweet potato tissues, and these expression differences provide important clues for further screening and identifying key candidate genes involved in sweet potato response to HM stress.

### 3.7 Expression patterns of IbABC family genes under iron, aluminum, zinc, and copper stress

**Figure 8:**
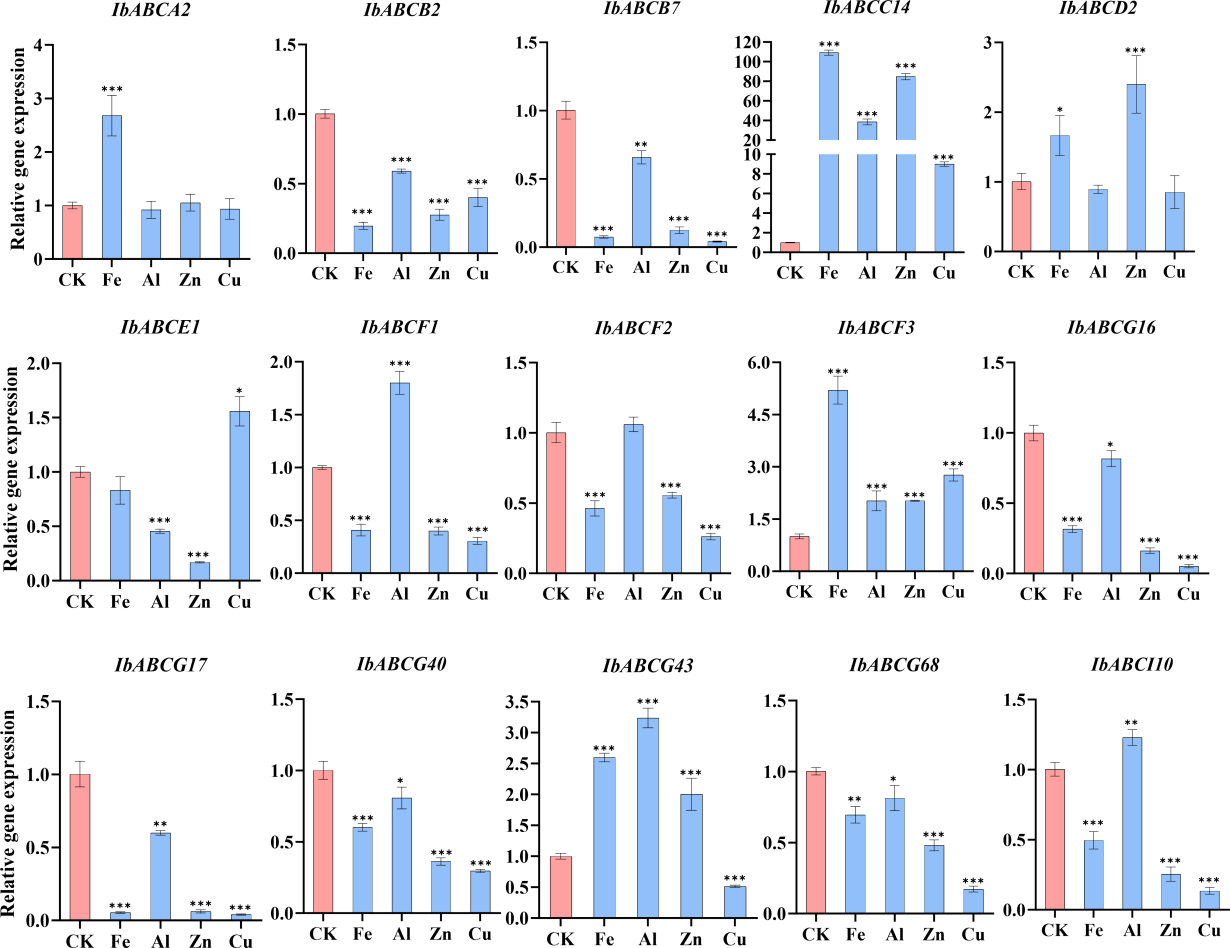
qRT-PCR analysis of candidate genes from the *IbABC* gene family under 10 days of iron (Fe), aluminum (Al), zinc (Zn), and copper (Cu) stress. Data are presented as mean ± SD (n=3). Red indicates CK (control); blue indicates treatment. Asterisks denote significant differences: * *P* < 0.05; ** *P* < 0.01; *** *P* < 0.001.

To investigate the potential roles of the *IbABC* gene family in response to HM stress, qRT-PCR analysis was conducted on 15 candidate genes (**Figure 8**). The results showed that *IbABCB2*/*7* and *IbABCG16*/*17*/*40*/*68* were consistently downregulated under Fe, Al, Zn, and Cu stress. Conversely, *IbABCA2* was specifically upregulated under iron stress, while *IbABCF1* and *IbABCI10* exhibited significant induction under aluminum stress. Notably, *IbABCG43* showed an upregulated pattern under Fe, Al, and Zn stress but was downregulated under Cu stress; this differential expression profile likely reflects an adaptive regulatory strategy to different HMs. Most significantly, *IbABCC14* was strongly induced by all four HM treatments, with expression levels increasing approximately 109.15-fold, 38.57-fold, 84.68-fold, and 9.00-fold under Fe, Al, Zn, and Cu treatments, respectively, suggesting its role as a pivotal regulator of HM transport in sweet potato. Given its constitutive high expression in fibrous roots and its sustained, significant upregulation under multiple HM stresses, *IbABCC14* likely plays a crucial functional role in the transport and homeostasis of Fe, Al, Zn, and Cu.

**Figure 9:**
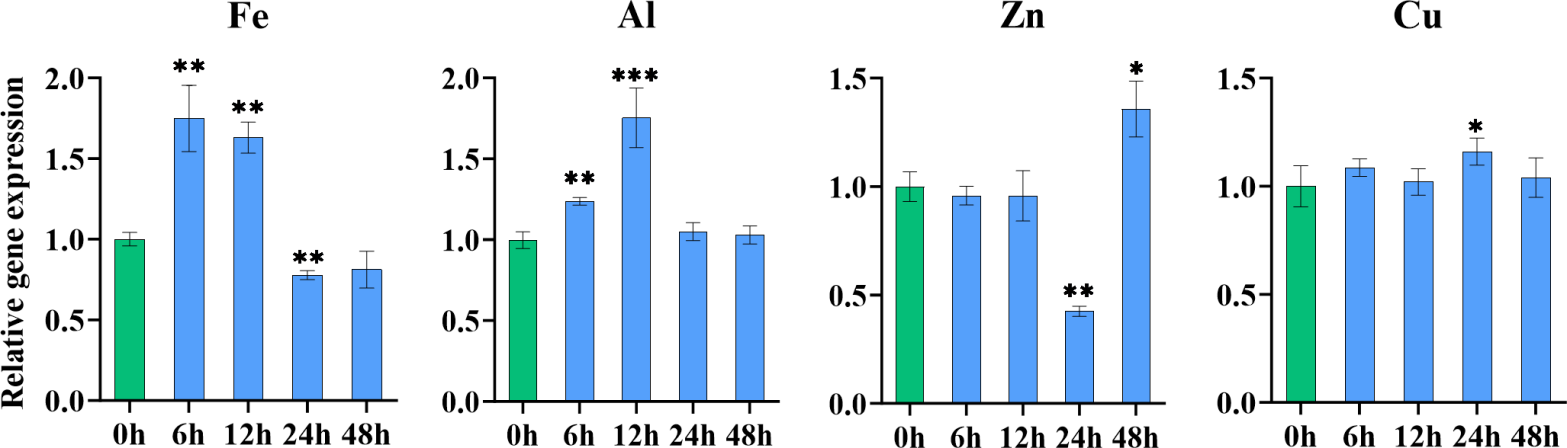
qRT-PCR expression analysis of *IbABCC14* at 0 h, 6 h, 12 h, 24 h, and 48 h under iron (Fe), aluminum (Al), zinc (Zn), and copper (Cu) stress. Data are presented as mean ± SD (n=3). Green bars represent the control group (0 h), and blue bars represent the treatment groups, displayed separately at different time points. Asterisks indicate significant differences compared with the control group: * *P* < 0.05; ** *P* < 0.01; *** *P* < 0.001.

Building on the significant differential expression of *IbABCC14* observed on day 10, this study further utilized quantitative real-time PCR (qRT-PCR) to analyze its expression dynamics during the early stages of stress, with detection time points at 0, 6, 12, 24, and 48 h (**Figure 9**). The results showed that compared to 0 h, the expression of *IbABCC14* under Fe and Al stress followed a trend of initial up-regulation followed by down-regulation, suggesting a rapid early response possibly followed by a regulatory recovery phase. Under Zn stress, the gene was down-regulated at 24 h and subsequently showed an up-regulatory trend at 48 h, indicating a potential delayed induction mechanism; conversely, under Cu stress, the expression of *IbABCC14* remained relatively stable across all time points, suggesting its involvement in the Cu stress response in a steady-state manner. Notably, the magnitude of expression changes at all early time points was significantly smaller than that observed on day 10. In conclusion, within the first 48 hours of HM stress, the expression changes of *IbABCC14* were limited-far below the induction levels observed on day 10-showing only slight fluctuations under Fe, Al, and Zn stress and remaining almost entirely stable under Cu stress, which indicates that *IbABCC14* is not an early transient response gene but rather primarily participates in HM transport and homeostasis regulation during the middle and late stages of stress.

### 3.8 Correlation between IbABC gene expression levels and agronomic traits and homologous gene analysis

**Figure 10:**
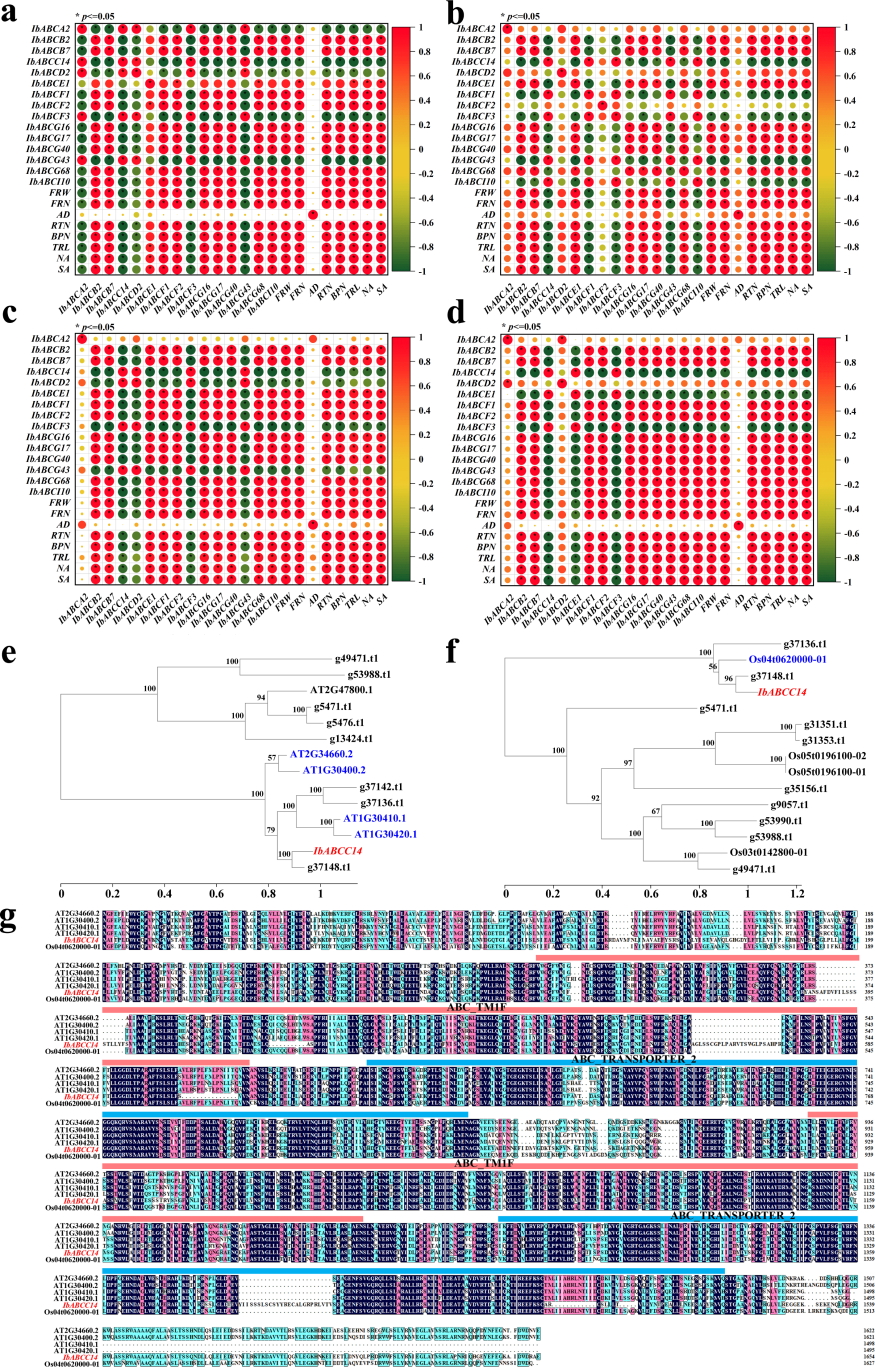
A heatmap showing the correlation between *IbABC* candidate gene expression levels and agronomic traits. An asterisk in the heatmap indicates a significance level of P < 0.05 (*). FRW, fresh root weight; FRN, fresh root number; AD, average diameter; RTN, root tips number; BPN, branch points number; TRL, total root length; NA, network area; SA, surface area. **(a)** Correlation analysis of gene expression and agronomic traits under iron (Fe) stress. **(b)** Correlation analysis of gene expression and agronomic traits under aluminum (Al) stress. **(c)** Correlation analysis of gene expression and agronomic traits under zinc (Zn) stress. **(d)** Correlation analysis of gene expression and agronomic traits under copper (Cu) stress. **(e)** Homology comparison of *IbABCC14* with Arabidopsis genes. **(f)** Homology comparison of *IbABCC14* with rice genes. **(g)** Multiple sequence alignment of homologous genes.

To investigate the correlation between *IbABC* gene expression and agronomic traits under HM stress, a Pearson correlation analysis was conducted using data collected 10 days after treatment with Fe, Al, Zn, and Cu. The expression of specific *IbABC* genes showed significant positive or negative correlations (*P < 0.05) with two key indicators of root development: fibrous root fresh weight and root number. Under Fe stress, the expression of *IbABCB2*/*7*, *IbABCF1*/*2*, *IbABCG16*/*17*/*40*/*68*, and *IbABCI10* was significantly positively correlated with these traits, whereas *IbABCA2*, *IbABCC14*, *IbABCF3*, and *IbABCG43* exhibited significant negative correlations. Similar patterns were observed under Al stress, where *IbABCE1*, *IbABCB2*/*7*, and *IbABCG16*/*17*/*40*/*68* showed positive correlations, while *IbABCC14*, *IbABCF1*/*3*, *IbABCG43*, and *IbABCI10* were significantly negatively correlated (**Figures 10a, b**). Under Zn stress, the composition of positively correlated genes (*IbABCB2*/*7*, *IbABCF1*/*2*, *IbABCG16*/*17*/*40*/*68*, *IbABCI10*) and negatively correlated genes (*IbABCC14*, *IbABCF3*, *IbABCG43*) matched those identified under Fe stress (**Figure 10c**). Under Cu stress, *IbABCB2*/*7*, *IbABCF1*/*2*, *IbABCG16*/*17*/*40*/*43*/*68*, and *IbABCI10* again displayed significant positive correlations, while only *IbABCC14*, *IbABCF3*, and *IbABCE1* were negatively correlated (**Figure 10d**). In summary, these results indicate that sweet potato root development, as reflected by fibrous root fresh weight and root number, is strongly influenced by HM stress and closely associated with the expression patterns of specific *IbABC* genes, which may control root morphogenesis and ultimately influence yield determination by regulating the uptake and transport of metal ions.

To further evaluate the homology and potential function of *IbABCC14*, Sequence alignment revealed that *IbABCC14* shares four conserved domains with its homologous genes from *Arabidopsis thaliana* and rice. This result not only confirms their homology but also demonstrates the high evolutionary conservation of their core functional regions (**Figure 10g**). Using TBtools, sequence homology alignment with *Arabidopsis thaliana* genes was performed using TBtools. The results showed that *IbABCC14* is highly homologous to A. thaliana AT2G34660.2 (*AtABCC2*), AT1G30400.2 (*AtABCC1*), AT1G30410.1 (*AtABCC12*), AT1G30420.1 (*AtABCC11*) (**Figure 10e**) as well as rice Os04t0620000-01 (**Figure 10f**). Previous studies have shown that *Arabidopsis* mutants lacking *AtABCC1* and *AtABCC2* exhibit strong sensitivity to arsenic, while heterologous expression of these transporters in yeast enhances arsenic tolerance (Song et al., 2010). Additionally, single and double knockout mutants of *AtABCC1* and *AtABCC2* display hypersensitivity to Cd stress, whereas overexpression confers increased Cd tolerance (Park et al., 2012). In rice, low arsenic accumulating varieties possess a shared SNP (single nucleotide polymorphism) within Os04t0620000-01 (Das et al., 2021).These findings support the hypothesis that *IbABCC14* plays a key role in mediating sweet potato responses to HM stress.

## 4 Discussion

HM pollution exhibits significant environmental persistence, triggering continuous ecological degradation and posing a severe threat to plant growth and ecological security, These metal ions are readily absorbed by crops, where they inhibit root development, disrupt nutrient uptake, and ultimately reduce yield and quality, potentially causing plant death (Hu et al., 2024). Consequently, understanding the adaptive and regulatory mechanisms underlying plant responses to HM stress has become a critical area of research (Guan et al., 2023). Among the mechanisms involved, ATP-binding cassette (*ABC*) proteins are essential transporters that participate in crucial biological processes, such as the CO_2_ concentrating mechanism, lipid biosynthesis, and HM sequestration (Li et al., 2022). While the *ABC* gene family has been extensively studied in several plant species like *Arabidopsis thaliana*, rice, *maize*, and tea plants, the functional roles of *ABC* genes in sweet potato, a high-biomass tuber crop with significant soil contact, remain largely unexplored. This study presents a comprehensive identification and analysis of the *ABC* gene family in sweet potato, examining its evolutionary relationships and expression patterns under HM stress caused by Fe, Al, Zn, and Cu. These findings provide valuable insights into the roles of *IbABC* genes in sweet potato’s response to HM stress and lay the foundation for developing molecular strategies for breeding HM-tolerant sweet potato cultivars. Previous studies have reported 129, 121, 133, and 170 *ABC* family members in *A. thaliana* (Sánchez-Fernández et al., 2001), rice (Garcia et al., 2004), *maize* (Guo et al., 2022), and tea plant (Shen and Li, 2023), respectively. While in our study 139 *IbABC* genes were count, sweet potato is comparable to maize, possessing more *ABC* genes than *Arabidopsis* and rice but fewer than tea plants. The differences in gene count between sweet potato and the Poaceae species (maize and rice) likely reflect lineage-specific expansion following the divergence of monocots and dicots.

Further studies on subcellular localization predictions revealed that most *GmABC* proteins in soybean and *HvABC* proteins in barley localize to the plasma membrane, with smaller proportions found in mitochondria, vacuoles, the nucleus, the endoplasmic reticulum, and the Golgi apparatus (Huang et al., 2021). A similar distribution pattern was observed in our study, where the majority of *IbABC* proteins were predicted to be localized in the plasma membrane. Additionally, other *IbABC* proteins were distributed across chloroplasts, the Golgi apparatus, the nucleus, mitochondria (Zhang et al., 2020), and vacuoles. Phylogenetically, previous studies have divided *ABC* gene families of *Arabidopsis*, tea *plant*, and several Gossypium species (Cui et al., 2021) into eight subfamilies. Consistent with these findings, sweet potato *ABC* gene family was also classified into eight subfamilies (**Figure 1a**). Gene structure and conserved motif analyses further supported functional diversification among subfamilies. Studies have found that both the *ABCG* and *ABCF* subfamilies within the barley *ABC* gene family contain a specific motif 4, which is likely an indispensable structural component for ensuring the normal function of specific proteins. The diversity of motif composition among different subfamilies may be an important factor contributing to their functional differentiation during evolution (Zhang et al., 2020). Similarly, in the pear *ABC* gene family, members originating from the same subfamily exhibit strong consistency in both sequence length and motif distribution patterns. The widespread presence of conserved motifs in *PbrABC* proteins suggests that their domains responsible for executing key functions maintain a high degree of conservation throughout evolutionary history (Kou et al., 2024). Earlier studies reported 10 conserved motifs in *ABC* gene families of pepper (Lopez-Ortiz et al., 2019) and lentil (Singh et al., 2024). In these species, *ABCG* subfamily members typically contained Motifs 1, 2, 4, and 7, whereas *ABCB* subfamily members predominantly possessed Motifs 1, 2, 5, and 6. A similar motif distribution pattern was observed in the present study, where members within each *IbABC* subfamily displayed highly conserved structural and motif features, indicating potential functional differentiation of this gene family among different subgroups. Motif 3 is present in most *LcABC* proteins, contains the characteristic sequence of the *ABC* gene family, and is classified as a type 1 export protein. Meanwhile, motifs 4 and 7, which are commonly found in *LcABC* proteins, correspond to an *ABC* transporter-like structure and a P-loop nucleoside triphosphatase (NTPase), respectively (Lopez-Ortiz et al., 2019). Our study found that motifs 1, 3, and 7 occur with high frequency in the *ABCB* and *ABCC* subfamilies, suggesting that they may play important roles in substrate transport processes. Previous studies have shown that motif 8 represents the *ABC*-type lipoprotein export system, which plays a key role in defending against toxic substances (such as antibiotics) and maintaining viability. Members of the *ABCG* subfamily in almond exhibit eight distinct motif types, namely motifs 1, 2, 3, 4, 5, 7, 8, and 9. Our study results are similar to those findings in almond, with motif 8 occurring at a high frequency, suggesting that it may play a role in defending against HM stress (Zhang et al., 2024).

Gene family expansion mechanisms differ across species. While tandem duplication drives *ABC* gene expansion in lentil, transposition duplication predominates in sweet potato, likely due to its complex genomic architecture. Additionally, the sweet potato *ABC* gene family shows a closer evolutionary relationship with dicots than monocots, like the differentiation observed between *Arabidopsis thaliana* and rice (Zhang et al., 2024). At the transcriptional regulatory level, cis-acting elements are crucial for regulating gene expression in response to environmental stress (Passricha et al., 2017). Previous research identified nine representative cis-acting elements in maize *ABC* gene promoters and 14 cis-acting element types in the *Artemisia annua ABC* subfamily *AarPDRs*, associated with hormone response, light response, and stress signaling (Han et al., 2025). In this study, we identified 66 cis-acting elements in the promoter regions of *IbABC* genes, categorized into four main groups: light-responsive, growth and development-related, phytohormone-responsive, and stress-responsive elements. These findings are consistent with previous reports on wheat *ABC1K* genes (Gao et al., 2022). Similarly, this study found that the promoter regions of the sweet potato *IbABC* gene family are also rich in cis-acting elements such as Box 4, MYC, and MYB. These elements are important in regulating gene expression and influencing plant growth, development, and stress resistance. Notably, within the entire family, only *IbABCB5* and *IbABCF3* were found to lack MYC elements, although they still contain abundant MYB and Box 4 elements. This result further reveals that MYC elements are relatively conserved in the *IbABC* gene family.

Previous research based on RNA-Seq data has shown that members of the flax *ABC* gene family, such as *LuABC* and *LuHMA*, are highly expressed in roots and seeds (Khan et al., 2020). In alfalfa, certain *MsABC1K* genes, including *MsABC1K1*/*3a*/*3b*/*4*/*5a*/*6*/*7*/*8a*/*8b*/*9* are mainly expressed in leaves and flowers, while *MsABC1K5b*/*5c*/*12*/*15* are primarily expressed in stems and leaves. Similarly, in wheat, *TaABC1K3* and *TaABC1K6* show the highest expression in leaves (Chen et al., 2024). Additionally, *TaABC1K3* also shows high expression in developing grains, while *TaABC1K6* is expressed at lower levels in roots, stems, and developing grains. Consistent with these findings in other species, *IbABC* gene family in sweet potato also demonstrates clear tissue-specific expression patterns. For example, *IbABCF1/2/3*, *IbABCB4/6*, *IbABC18*, *IbABD1*, and *IbABCC14* showed high expression across all tested organs in ‘Yan 25’. Notably, *IbABCG6/17/68/43*, *IbABCF3/2*, and *IbABCC14* also exhibited high expression in all three root types of ‘Annayu’, suggesting potential roles in root development and root-specific physiological processes. suggesting that these genes may play important roles in root development or root specific physiological functions. Previous studies have highlighted that many *ABC* genes show specific responses to metal ions, providing valuable insights into the physiological roles of these genes. For example, in *Arabidopsis*, the *ABC* transporter *AtPDR12* is strongly upregulated under lead stress, where it facilitates the efflux of lead-associated toxic compounds, enhancing root length and plant fresh weight, thus improving lead tolerance (Lee et al., 2005). In rice, *OsPDR20* is strongly induced by cadmium stress, and RNAi-mediated downregulation of this gene inhibits root and shoot growth, reduces biomass accumulation, and lowers chlorophyll content, indicating its role in cadmium accumulation and homeostasis (Gao et al., 2022). Similarly, *AtATM3* in *Arabidopsis* functions as a positive regulator of cadmium resistance, as its overexpression enhances tolerance by reducing non-protein thiol accumulation under cadmium stress, despite showing no phenotypic differences under normal conditions (Kim et al., 2006).

Functional studies in legumes support the tissue-specific expression patterns of *ABC* genes in stress responses. For instance, overexpression of *GsABCI1* in soybean significantly increases root length under aluminum stress, improving aluminum tolerance compared to the wild type (Zhao et al., 2024). Similarly, overexpression of *GmABCG5* enhances tolerance to iron deficiency, whereas its suppression reduces tolerance. In poplar, *PtABCC1* promotes mercury transport from roots to aerial tissues, and overexpression in both *Arabidopsis* and poplar increases mercury tolerance (Sun et al., 2018). Additionally, silencing of *HvABCB25* in barley increases sensitivity to aluminum stress and restricts root growth, while its overexpression enhances relative root growth and dry weight under aluminum stress (Liu et al., 2021). This study used RT-qPCR to detect the expression patterns of *IbABC* family genes in sweet potato vine cuttings under Fe, Al, Zn, and Cu stress. After 10 days of treatment (**Figure 8**), the 15 genes exhibited diverse and complex expression trends. Notably, *IbABCC14* showed sustained and significant upregulation under all four HM stresses, with expression levels increasing by approximately 109.2-fold, 38.6-fold, 84.7-fold, and 9.0-fold, respectively. Furthermore, at different time points of 0, 6, 12, 24, and 48 hours, *IbABCC14* also exhibited diverse expression patterns under the four HM stresses (**Figure 9**). Previous studies have shown that plant *ABC* genes generally exhibit dynamic and differential expression patterns under HM stress. For instance, *ZmABC* genes display diverse response characteristics to lead stress (Xie et al., 2012) and *PtoABCG36* is induced by cadmium, reaching its expression peak 12 hours after treatment (Wang et al., 2019). The expression of *SsABCc3*, *SsABCc9*, *SsABCc11*, *SsABCd1*, and *SsABCf1* in *Salvia sclarea* changes progressively after 1, 5, and 10 days of manganese treatment, with the highest expression observed on day 10 (Liaquat et al., 2022). These findings collectively indicate that plants respond to abiotic stress through temporally specific transcriptional regulation during dynamic growth processes.

This study found that multiple *IbABC* genes in sweet potato exhibited diverse expression patterns during the 10-day HM stress treatment, among which the unique expression characteristics of *IbABCC14* were particularly critical, suggesting that it may be a core regulatory factor in sweet potato’s response to multiple HM stresses. Previous studies have shown that the absence of ABCC-type transporters, like *AtABCC1* and *AtABCC2* in *Arabidopsis*, makes plants highly sensitive to arsenic, while their heterologous expression in *Saccharomyces cerevisiae* enhances arsenic tolerance and accumulation (Song et al., 2010). Research in rice identified a single nucleotide polymorphism (SNP) associated with low arsenic accumulation in 19 aromatic accessions, which shares homology with *IbABCC14* (Das et al., 2021). Our findings confirm that *IbABCC14* is highly homologous to these transporters, with amino acid sequence alignment revealing conserved domains. Moreover, the promoter region of *IbABCC14* is enriched with cis-acting elements like G-box and STRE, which are essential for regulating plant gene transcription. These results highlight the crucial role of *IbABCC14* in responding to HM stress and underscore its potential for improving stress tolerance in sweet potato.

Many *ABCC* proteins, including *AtABCC1* and *AtABCC2*, are tonoplast-localized transporters responsible for sequestering toxic metals such as arsenic and cadmium into the vacuole, playing a key role in plant detoxification (Park et al., 2012). In this study, we observed that *IbABCC14* shares high homology with these proteins and exhibits strongly induced expression under HM stress, particularly under iron stress. However, its expression pattern diverges from the classical role of *AtABCC1/2*, which are primarily involved in arsenic/cadmium detoxification. In rice, the *ABC* gene family member *ARG1* regulates the transport and homeostasis of cobalt (Co) and nickel (Ni) in chloroplasts, preventing excessive Co and Ni from interfering with essential metal cofactors in chlorophyll and key metalloproteins (Li et al., 2020). This suggests an emerging role for *ABCC* subfamily members not only as detoxifiers of xenotoxic substances but also as regulators of essential metal homeostasis, such as iron. Based on these analyses, *IbABCC14* is hypothesized to participate in the vacuolar transport of HM–ligand complexes, particularly phytochelatin complexes. However, this hypothesis requires further experimental validation. In sweet potato, it likely acts as a key tonoplast transporter involved in the sequestration of these complexes. While the typical phytochelatins pathway is primarily associated with the detoxification of non-essential HMs like cadmium and arsenic, recent studies suggest that under iron-excess-induced oxidative stress, cytoplasmic iron ions may bind with glutathione (GSH) or other thiol ligands to mitigate Fenton reaction-induced oxidative damage. The strong upregulation of *IbABCC14* under iron stress is likely to promote the transport of iron-thiol complexes (e.g., Fe-GSH or Fe-PCs) into the vacuole, thereby maintaining cytoplasmic homeostasis and protecting cellular structures from oxidative damage (Röhricht et al., 2023; Weber et al., 2023). This hypothesis not only explains *IbABCC14* specific response to Fe stress but also provides a unified functional interpretation for its broad induction under other HM stresses such as Al, Zn and Cu. While this hypothesis requires further validation through plant genetic transformation, protein subcellular localization, and interaction gene verification, it identifies *IbABCC14* as an important candidate gene for improving HM tolerance in crops. Moreover, this study provides new insights into the molecular mechanisms of *ABC* transporters in regulating the cross-talk between HM stress response and metal homeostasis.

## 5 Conclusions

In this study, a total of 139 *IbABC* genes were identified across the 15 chromosomes of sweet potato and classified into eight subfamilies, characterized by conserved structural motifs and stress related cis-elements. Expression analysis revealed distinct tissue-specific patterns, with 15 genes showing differential expression under HM stress. Notably, *IbABCC14* was significantly up-regulated in response to Fe, Al, Zn, and Cu treatments. These findings provide promising candidate genes for enhancing HM tolerance in crops, with functional validation through transgenic approaches and gene editing to follow.

## Data Availability

The original contributions presented in the study are publicly available. This data can be found here: https://ngdc.cncb.ac.cn/, PRJCA036206.
