# Supplementary figures and images for: Genome-wide identification of the ABC gene family in sweet potato and its expression profiles in response to iron, aluminum, zinc, and copper stresses
Source: Front Plant Sci. 2026 Jun 10;17:1815302. doi: 10.3389/fpls.2026.1815302 (PMC13291858; doi:10.3389/fpls.2026.1815302)

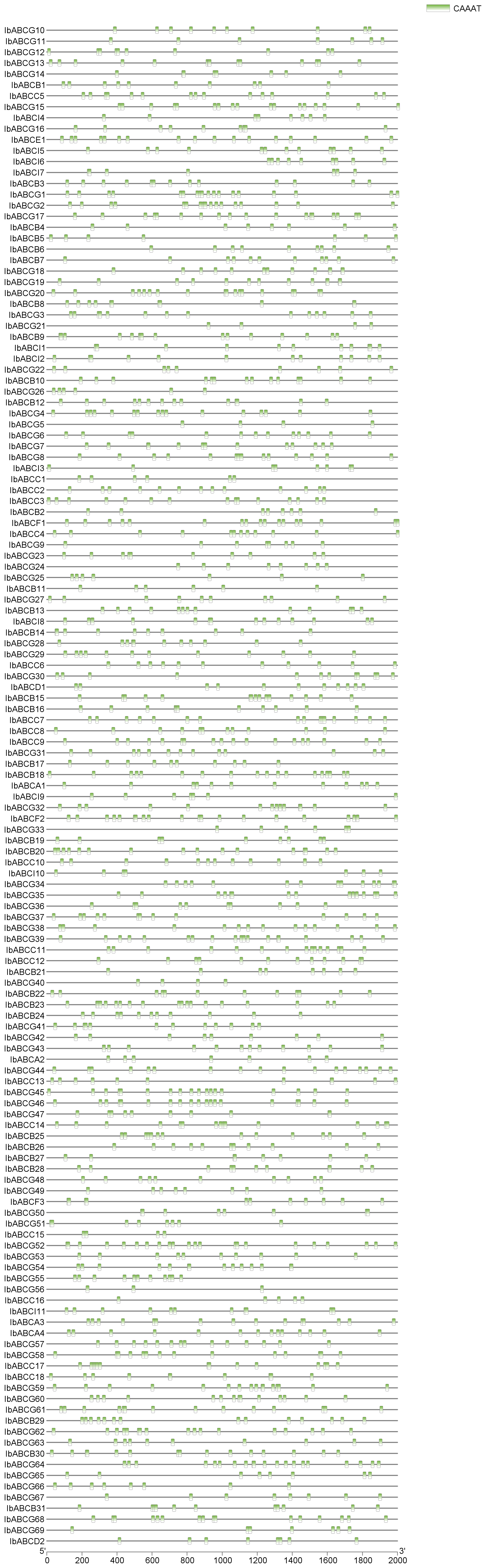

Supplement: Supplementary file 3 [file Image1.tiff]
